# The existing characteristics, transport patterns, and significance of nanoparticulate pollutants in groundwater surrounding an industrial park

**DOI:** 10.1371/journal.pone.0321264

**Published:** 2025-04-03

**Authors:** Guai Hu, Zeyu Wang, Yixiang Xie, ZiXia Lin

**Affiliations:** 1 School of Tourism and Geography, Shaoguan University, Shaoguan, China; 2 Shenyang Center of Geological Survey, China Geological Survey, Shenyang, China; 3 Northeast Geological S&T Innovation Center of China Geological Survey, Shenyang, Liaoning Province, China; 4 Testing Center, Yangzhou University, Yangzhou, China; National Chung Cheng University, Taiwan & Australian Center for Sustainable Development Research and Innovation (ACSDRI), Australia

## Abstract

Nanoparticles, increasingly recognized by regulators and the public, have become potential pollutants in various media, including water. Water security, a pressing global issue, is exacerbated by industrialization, with industrial wastewater being a significant contributor to water pollution. This study collected nanoparticles from groundwater samples in residential areas surrounding the Mingzhu Industrial Park in Guangdong Province, China. To understand their characteristics as potential pollutants in wastewater and the information they provide, field emission transmission electron microscopy (TEM) was used to analyze the composition, elemental content, morphology, and crystal structure of the nanoparticles. The nanoparticles originated from three sources: factories, water pipes, and the natural environment. They aggregate and oxidize during transport, ensuring their stability in water. This study elucidates the characteristics and transport patterns of particulate pollutants in groundwater near industrial parks, a prerequisite for assessing the hazards of nano-pollutants in natural environments. Additionally, it provides valuable insights for future discussions on their ecological impacts and advancements in water detection and treatment technologies.

## 1. Introduction

The world is experiencing severe water scarcity due to industrialization and urbanization [1,2]. Over 4 billion people face severe water scarcity, and more than half of the global population may be water insecurity [3]. Industrial wastewater, comprising more than 50% of total wastewater [4], is a primary concern. To address the issue of severe water pollution, various wastewater treatment methods have been developed. Researchers have proposed different filtration techniques for industrial wastewater, including precipitation [5–7], filtration [8], air flotation [9], coagulation [10], adsorption [11,12], electrochemical techniques [13–17], chemical neutralization [18–20], and disinfection [21]. These methods primarily focus on specific elements or substances but tend to overlook the forms in which pollutants exist. It would be more reasonable and effective to adopt different treatment methods for different forms of pollutants, which requires a deeper understanding of the states of pollutants in real-world water environments.

Nanoparticles are emerging pollutants with unique properties, such as the ability to travel long distances [22–26]. Additionally, nanoparticles can directly damage cells due to their small size, leading to distinct nanoparticle toxicity [26]. This toxicity includes genetoxicity caused by DNA damage and biological toxicity resulting from damage to cell membranes, mitochondria, and other cellular structures [26–31]. Recent studies have shown that the elemental composition of nanoparticles found in areas with industrial activity, urbanization, and mining deposits is closely linked to the elements commonly found in these environments [22–24,32–34]. However, the exact sources and characteristics of these nanoparticles remain unclear. The presence of multiple sources of nanoparticles in the same medium is also uncertain, posing challenges for researchers to further analyze their harmful effects and develop effective treatment methods. Moreover, current toxicological research on nanoparticles is primarily based on laboratory data, which may not fully reflect their harmfulness in real-world environments. Verifying the occurrence of nanoparticles in natural environments can help bridge the gap between laboratory research and environmental assessments.

To address this issue, this study collected groundwater samples from the Mingzhu Industrial Park and analyzed the occurrence and characteristics of the nanoparticles using field emission transmission electron microscopy (TEM), identifying potential sources. The results revealed three potential sources of nanoparticles near the park: factories, natural processes, and water pipes. Additionally, nanoparticles commonly form aggregates and undergo oxidation, which may enhance their stability. These findings contribute to a better understanding of the ubiquity of nanoparticles in natural environments and provide valuable data for future discussions on their ecological impact and the development of advanced water detection and treatment technologies.

## 2. Materials and methods

### 2.1. Study area

The Mingzhu Industrial Park, which has been in operation since 2003, is located in the Conghua District, Guangdong Province, China. It serves as a representative example of a subtropical monsoon climate zone, characterized by an average annual precipitation of 1,783 mm. The region features a well-developed network of rivers and lakes, highlighting the importance of water security in such areas. With over 130 enterprises operating within its boundaries, the Mingzhu Industrial Park primarily focuses on the auto parts, biomedicine, new energy, and new materials industries [35]. Pollutants produced by industrial activities can spread into the surrounding environment [36–38]. Sewage discharge is managed in accordance with the “Surface Water Environmental Quality Standards” and “Surface Water Class V Quality Standards”. There are few specific management in controlling nanoparticulate pollutants and still cases of excessive pollution [39]. As the sole industrial park in the vicinity and the environment is relatively independent, this area offers a unique opportunity for research into the sources of nanoparticles without interference from other industrial pollutants.

### 2.2. Sample collection and analysis methods

**Fig 1:**
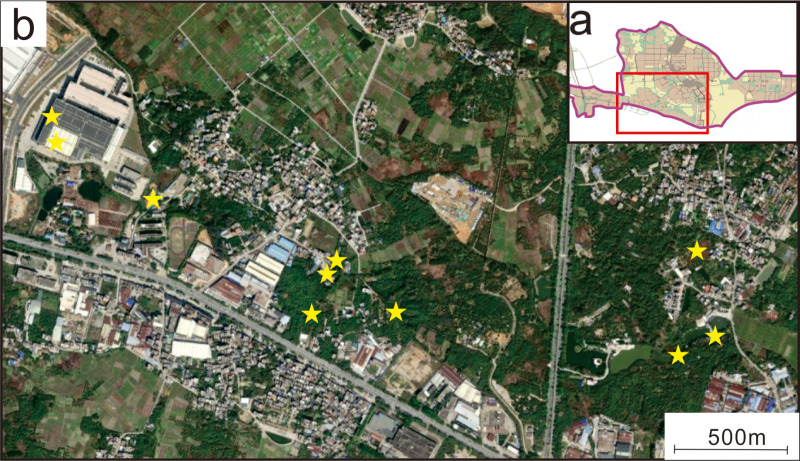
Sampling map of the Mingzhu Industrial Park: (a) Industrial Park overall sketch map of the Mingzhu Industrial Park; the red frame is the area in Figure b; (b) satellite map of the Mingzhu Industrial Park; the yellow star is the location where the groundwater sample was collected.

Samples were collected from rural settlements near the Mingzhu Industrial Park (Fig 1) to assess the quality of the water bodies. Groundwater was sampled from taps and wells in the study area in July (high water season) and January (low water season), respectively, totaling ten sampling locations [39,40]. Sampling was performed when there was no rain to prevent precipitation affecting the composition of the particles. These water sources are directly linked to groundwater and remain untreated during the process of being pumped from underground to the surface for use, which accurately reflects the water conditions around the industrial park. Furthermore, the collected water represents the domestic supply for residents, providing insight into their water safety.

We used polyethylene bottles cleaned with ultrapure water for water sampling to ensure a contamination-free collection process. After collecting the groundwater sample, it was poured into a beaker. A transmission electron microscope grid was then immersed in the water samples for 30 minutes to adsorb the nanoparticles. The grid was placed in a special box and sent to the laboratory for testing. All instruments used in this process were cleaned with ultrapure water to prevent contamination [26,41].

The samples were analyzed at Yangzhou University’s Testing Center using transmission electron microscope (TEM) equipped with high-resolution transmission electron microscope (HRTEM), selected-area electron diffraction (SAED), and X-ray energy-dispersive spectrometer (EDS). The instrument model is Tecnai G^2^ F30-Twin TEM, which had a resolution of 0.17 nm and a maximum acceleration voltage of 300 kV. Morphological characteristics such as category, size, and shape were analyzed using TEM, while chemical composition was semi-quantitatively analyzed using EDS. The crystal lattices of the samples were analyzed using SAED and HRTEM. The test data was analyzed and processed by Digital Micrograph 3.7.4 and TEM Imaging & Analysis.

## 3. Result

### 3.1. Particles with Fe or Zn as the main component

**Fig 2:**
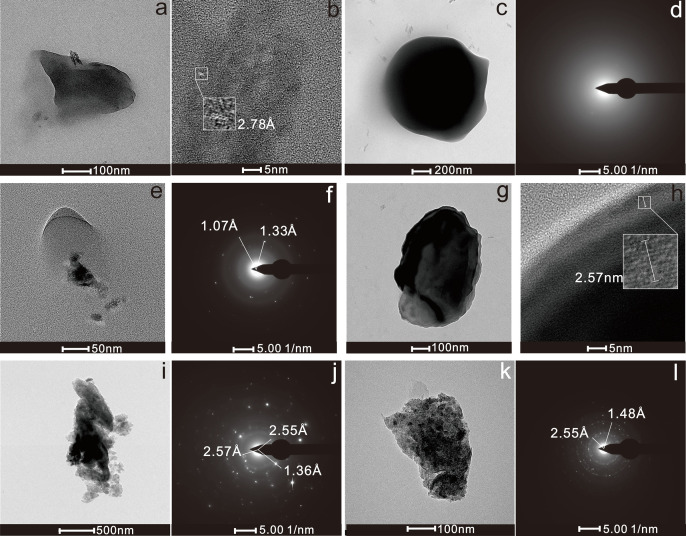
Fe-Zn oxide particles in groundwater: (a) TEM image of ID1; (b) HRTEM image of ID1; (c) TEM image of ID2; (d) electron diffraction pattern of ID2; (e) TEM image of ID3; (f) electron diffraction pattern of ID3; (g) TEM image of ID4; (h) HRTEM image of ID4; (i) TEM image of ID5; (j) electron diffraction pattern of ID5; (k) TEM image of ID6; (l) electron diffraction pattern of ID6.

Fe and Zn are the predominant elements in the groundwater samples collected from the Mingzhu Industrial Park and are also the main elements in the galvanized water pipes commonly found around the park. Four representative Zn-O particles (ID1–ID4) were examined morphologically, crystallographically, and elementally (Table 1). The particle sizes ranged from 38.33 to 1,244 nm, with a predominance of irregular shapes between 200 and 400 nm (Fig 2a, Fig 2c, Fig 2e, Fig 2g). Most particle boundaries were clear, except for ID2, which appeared fuzzy. Crystal structures with distinct diffraction spots or lattice patterns were observed in ID1, ID3, and ID4. The lattice spacings were 2.78 Å (1,1,0), 1.07 Å (2,0,3), 1.33 Å (2,0,1), and 2.57 Å (0,0,2), corresponding to ZnO (PDF074-9942) crystal faces (Fig 2b, Fig 2f, Fig 2h). In contrast, ID2 was amorphous, with weak diffraction spots and no diffraction rings (Fig 2d). The elemental composition revealed that the particles were primarily composed of Zn (8.2–83.3 wt%), O (16.2–58.1 wt%), and minor amounts of Na (13.2 wt%), Al (1.3–7.8 wt%), Si (0.4–2.4 wt%), and Fe (0.5–5.4 wt%). Overall, ID1–ID4 were identified as Zn-O particles. Two Fe oxide particles, ID5 and ID6, were selected for analysis. Their sizes ranged from 253 to 1,590 nm (Fig 2i, Fig 2k), with irregular shapes and high-contrast boundaries. Both particles were primarily polycrystalline. Lattice spacing measurements revealed that the lattice spacings of ID5 and ID6 were 2.55 Å and 1.48 Å (Fig 2j, Fig 2l), corresponding to the (1,1,0) and (3,3,0) crystal planes of Fe_2_O_3_ (PDF088–2359), respectively. Additionally, ID5 had lattice spacings of 1.36 Å and 2.57 Å, which aligned with the (1,1,2) and (0,0,2) crystal faces of ZnO (PDF074–9942). Chemically, ID5 was composed of Zn (11.7 wt%), Fe (2.6 wt%), O (76.9 wt%), Na (4.7 wt%), Al (2.3 wt%), and Si (1.8 wt%). In contrast, ID6 had a higher Fe content (47.4wt%) and lower O content (45.5 wt%), along with Al (1.0 wt%), Si (4.5 wt%), and a small amount of Zn (1.6 wt%). ID6 is a Fe-O particle, while the presence of Fe-Zn-O in ID5 suggests an aggregate structure consisting of Fe-O and Zn-O particles. The coexistence of Fe-Zn and Al-Si in these particles was observed.

**Table 1:** EDS results of nanoparticles in groundwater samples from the Mingzhu Industrial Park.

Number	Weight O%	Weight Fe%	Weight S%	Weight Na%	Weight Ti%	Weight Si%	Weight Cl%	Weight Ca%	Weight Al%	Weight Mg%	Weight Mn%	Weight Mo%	Weight Pb%	Weight Zn%	Weight Cr%	Weight Ba%	Weight Cu%	Weight W%	Main element composition
ID1	58.1	5.4		13.2		2.4	4.8		7.8					8.2					Zn-Fe-O
ID2	42.5					0.4			1.3	2.6				53.2					Zn-O
ID3	37													63					Zn-O
ID4	16.2	0.5												83.3					Zn-O
ID5	76.9	2.6		4.7		1.8			2.3					11.7					Fe-Zn-O
ID6	45.5	47.4				4.5			1					1.6					Fe-O
ID7	78.4		1.7			0.6	0.7										18.6		Cu-S-O
ID8	42.4		5.8			3			2.4							46.4			Ba-S-O
ID9	24.5		6.5			1.9			1.6							65.5			Ba-S-O
ID10	64	4	0.8					1.8		2.6					26.8				Cr-O
ID11	4.9		31.1									64							Mo-S-O
ID12	40.8	14			2.5	3.5			4.2		23.3		11.7						Pb-Mn-O
ID13	36.7	2.1			25.9				3									32.3	Ti-W-O
ID14	52.8	1.8			37.2	0.4			2.7					5.1					Ti-O
ID15	36.6	2.2		2.4	40.2	0.2			0.1					18.3					Ti-O
ID16	50.2	3			43.8	1.1			1.6	0.3									Ti-O
ID17	62.5	1.7		1.9	1.4	13.4			16.4					2.7					Al-Si-O
ID18	25.9	18.9		0.6		2.2			49						3.4				Al-O

### 3.2. Particles with heavy metals as the main components

**Fig 3:**
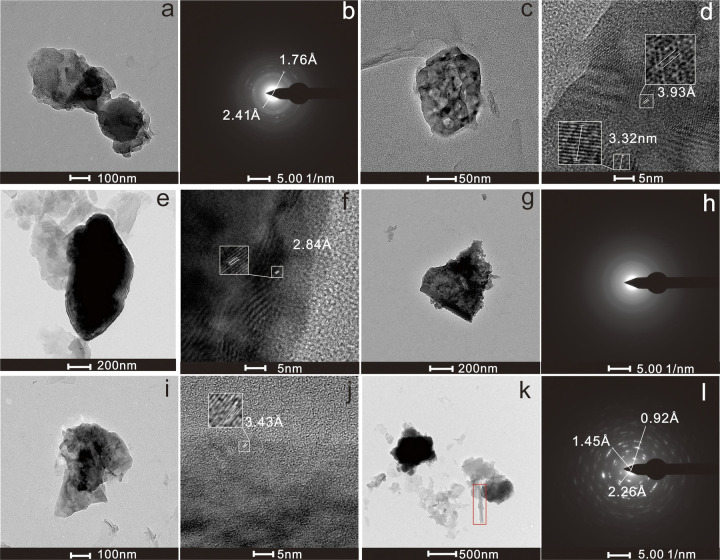
Heavy metal-bearing particles in groundwater: (a) TEM image of ID7; (b) electron diffraction pattern of ID7; (c) TEM image of ID8; (d) HRTEM image of ID8; (e) TEM image of ID9; (f) HRTEM image of ID9; (g) TEM image of ID10; (h) electron diffraction pattern of ID10; (i) TEM image of ID11; (j) HRTEM image of ID11; (k) TEM image of ID12, where tubular halloysite(red box) with low contrast is combined with Pb Mn-bearing nanoparticles; (l) electron diffraction pattern of ID12.

Ten representative heavy metal particles (ID7-ID16) were identified in groundwater nanoparticles from the Mingzhu Industrial Park, containing Ba, Cu, Cr, Mo, Pb, Mn, W, and Ti (Table 1). The particle size and shape of each element are described as follows:

The Cu particles (ID7) were irregularly shaped, with a size of 323 ×  732 nm and high contrast (Fig 3a). Lattice spacings of 1.76 Å and 2.41 Å (Fig 3b) correspond to the (3,1,4) and (0,0,2) crystal planes of CuSO₄ (PDF007–5093). The composition is primarily Cu (18.6 wt%), O (78.4 wt%), and S (1.7 wt%), with small amounts of Si (0.6 wt%) and Cl (0.7 wt%), suggesting that these particles are sulfate particles of Cu.

Ba Particles (ID8, ID9): These particles have an elliptical shape with a size range of 112–1143 nm and clear boundaries (Fig 3c, Fig 3e). Lattice spacings of 3.93 Å, 3.32 Å, and 2.84 Å correspond to the (1,1,1), (1,0,2), and (1,1,2) crystal faces of BaSO_4_ (PDF073–6180), respectively (Fig 3d, Fig 3f). The composition is primarily Ba (46.4–65.5 wt%), S (5.8–6.5 wt%), and O (24.5–64.0 wt%), with small amounts of Si (1.9–3.0 wt%) and Al (1.6–2.4 wt%). These particles are likely Ba sulfate.

Cr Particles (ID10): These particles had a size of 434 ×  641 nm with an irregular shape and clear boundaries (Fig 3g). Overall, they are amorphous (Fig 3h), with Cr (26.8 wt%), O (64.0 wt%), Fe (4.0 wt%), S (0.8 wt%), Ca (1.8 wt%), and Mg (2.6 wt%) as the elemental components. It is believed to be an oxide particle of Cr with a minor Fe oxide content.

Mo Particle (ID11): This particle has a size of 521 ×  358 nm, with general contrast and an irregular shape (Fig 3i). The crystal lattice of the particle is well defined, with a lattice spacing of 3.43 Å (Fig 3j) corresponding to the (−6,0,2) crystal plane of MoSO_4_ (PDF070–9533). The primary elements are Mo (64.0 wt%), S (31.1 wt%), and O (4.9 wt%), suggesting that it is a sulfate particle of Mo.

Pb-Mn Particles (ID12): It exhibits a particle size of 770 ×  619 nm with high contrast, irregular shapes, and clear boundaries (Fig 3k). Low-contrast halloysite can be observed on the side. The size of the polycrystalline rings in the diffraction pattern is 1.45 Å, corresponding to the (1,4,5) crystal faces of Mn_2_O_3_ (PDF071–3820). The other rings measured 0.92 Å and 2.26 Å, corresponding to the (6,3,5) and (2,2,2) crystal planes of Pb_3_O_4_ (PDF076–1799), respectively ((Fig 3l). The predominant elements are Pb (11.7 wt%), Mn (23.3 wt%), O (40.8 wt%), and Fe (14.0 wt%); small amounts of Al (4.2 wt%), Si (3.5 wt%), and Ti (2.5 wt%) are also present. It can be assumed that the presence of Pb-Mn-O in ID12 suggests an aggregate structure consisting of Pb-O and Mn-O particles, with a small amount of halloysite.

**Fig 4:**
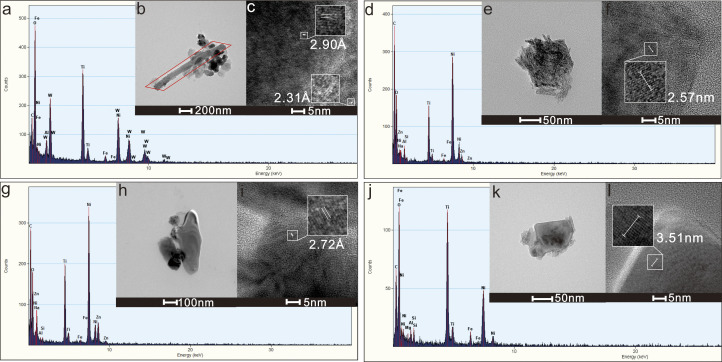
Heavy metal-bearing particles in groundwater: (a) EDX result diagram of ID13; (b) TEM image of ID13, the red box represents the halloysite; (c) HRTEM image of ID13; (d) EDX result diagram of ID14; (e) TEM image of ID14; (f) HRTEM image of ID14; (g) EDX result diagram of ID15; (h) TEM image of ID15; (i) HRTEM image of ID15;(j) EDX result diagram of ID16; (k) TEM image of ID16; (l) HRTEM image of ID16.

The W-Ti particle (ID13) has a diameter of 199 ×  372 nm, exhibiting high contrast, an irregular shape, and a clear boundary, and it forms an aggregation with halloysite (Fig 4b). The lattice spacing is 2.90 Å (Fig 4c), corresponding to the (2,1,1) crystal planes of TiO₂ (PDF071-4943), and 2.31 Å, corresponding to the (1,2,0) crystal plane of WO₃ (PDF087-2396). The main components are W (32.3 wt%), Ti (25.9 wt%), and O (36.7 wt%), with smaller amounts of Fe (2.1 wt%) and Al (3.0 wt%) also present. It is proposed that these particles consist mainly of W-O and Ti-O particles.

Ti particles (ID14, ID15, and ID16) range in size from 88 to 498 nm, exhibiting high contrast, irregular shapes, and clear boundaries (Fig 4b, Fig 4e, Fig 4h, Fig 4k). The measured lattice spacings are 2.90 Å, 2.57 Å, 2.72 Å, and 3.51 Å, corresponding to the (2,1,1) (0,0,2), (0,2,0), and (2,1,0) crystal planes of TiO_2_ (PDF071-4943), respectively (Fig 4c, Fig 4f, Fig 4i, Fig 4l). The major components are Ti (37.2 wt%-43.8 wt%), O (36.6 wt%-52.8 wt%), and Zn (0 wt%-18.3 wt%). Small amounts of Al (0.1 wt%-2.7 wt%) and Si (0.2 wt%-1.6 wt%) are also present. These particles are believed to be primarily composed of titanium oxide, possibly containing zinc oxide and minor clay mineral particles.

### 3.3. Particles of clay

**Fig 5:**
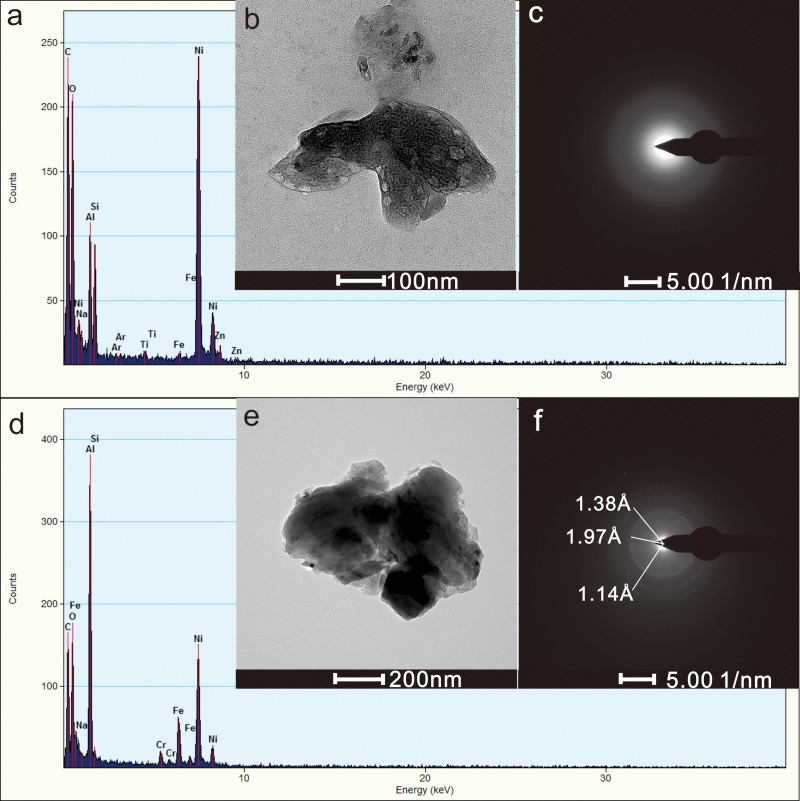
Clay particles in groundwater: (a) EDX result diagram of ID17; (b) TEM image of ID17; (c) electron diffraction pattern of ID17; (d) EDX result diagram of ID18; (e) TEM image of ID18; (f) electron diffraction pattern of ID18.

We observed clay particles, such as ID12 and ID13, in the results above. Two representative clay mineral particles, ID17 and ID18, were selected for detailed description. The ID17 particle had a size of 441 ×  266 nm and was amorphous with irregular shapes and clear boundaries (Fig 5b, Fig 5c). The main components were Al (16.4 wt%), Si (13.4 wt%), and O (62.5 wt%). Minor elements included Fe (1.7 wt%), Na (1.9 wt%), Ti (1.4 wt%), and Zn (2.7 wt%). The lamellar shape and Al-to-Si ratio suggest that it is kaolinite. The ID18 particle had a size of 595 ×  847 nm and exhibited an irregular shape with clear boundaries and moderate contrast (Fig 5e). The lattice spacings 1.14 Å, 1.38 Å, 1.97 Å (Fig 5f) correspond to the (-3,1,3), (3,3,1), and (2,2,1) crystal faces of Al(OH)_3_ (PDF077-0250). The main components were Al (49.0 wt%) and O (25.9 wt%) with minor amounts of Fe (18.9 wt%) and Cr (3.4 wt%). It is inferred that these particles are primarily gibbsite with trace amounts of Fe and Cr oxides.

## 4. Discussion

### 4.1. Elemental characteristics and sources of nanoparticles

These nanoparticles, which have a fixed element combination, often reflect the source of the particles, especially deposits and factories [33,42,43]. The manuscript speculates that the particles have three sources, based on the observed repeatable combination of elements: Zn-Fe particles from water pipes, heavy metal oxide/sulfate particles from industrial parks, and secondary mineral particles (mainly Al-Si-O) from the natural environment. The detailed description and source analysis is as follows: Zn-Fe nanoparticles, including ID1-ID6, primarily consist of Zn oxide with a minor Fe content (ID1-ID4) or Fe oxide with a trace amount of Zn (ID5-ID6). Fe and Zn frequently coexist, indicating a shared origin. The proportion of Fe-Zn particles (ID1-ID6) was comparable to that of other metallic elements (ID7-ID16). The Fe-Zn particles provided by the industrial park alone cannot reach this proportion. Additionally, the presence of numerous production industries in the park does not guarantee the coexistence of Fe and Zn in the particles. Therefore, factories may not be the main source of Fe-Zn particles. Wimmer et al. found a large number of Cu particles in the water of copper pipes [25]. In China, galvanized steel pipes are the primary material used for domestic water supply [44], although some water supply pipes are made of cement [45]. A significant number of galvanized steel pipes were observed at the outlets surrounding the Mingzhu Industrial Park. Galvanized steel pipes for water outlets can provide Fe and Zn. The dissolution and effects of water contribute to the corrosion of steel pipes [24,25,46,47]. Elements dissolved in water pipes can exist in the form of nanoparticles [24,25]. This is consistent with the phenomenon of Zn and Fe coexisting as nanoparticles observed in groundwater. The observed iron and zinc nanoparticles in water are likely formed by the corrosion of galvanized steel pipes, presenting a novel pollution source that warrants further investigation.

The second category of particles consists of nanoparticles composed of multiple heavy metals (ID7-ID16), such as Cu-S-O, Ba-S-O, Mo-S-O, Cr-O, Ti-O, Pb-Mn-Fe-O, and W-Ti-O. In the past, factory-associated nanoparticles have been found near industrial facilities [32,48–53]. The Mingzhu Industrial Park is the primary industrial park for automobiles and parts, biomedicine, new energy and materials, and fine chemicals [35]. Cu sulfate, barite (Ba sulfate), Mo disulfide, Cr, Ti oxide, and tungsten-titanium alloy (W-Ti) are commonly used as raw materials in vehicle manufacturing and the production of new materials. Pb-Mn metal precipitation is a common waste product of vehicle operation. The types and combinations of elements in the nanoparticles detected in this area correspond positively to the main industries in the industrial park. Additionally, the Mingzhu Industrial Park is remote, with no other sources of industrial pollution nearby. Besides the aforementioned heavy metal elements, Fe is also present in the particles; Fe is a commonly used element in industrial production and is the main component of water pipes. The Fe in the nanoparticles may originate from the Mingzhu Industrial Park or the corrosion of water pipes and is often associated with heavy metal-bearing particles. In general, the elemental composition of the heavy metal-bearing particles is closely related to that of the Mingzhu Industrial Park. The combination of elements in nanoparticles can reflect some of the materials found in industrial parks and can also be generalized to factories worldwide. Additionally, there are many Al-, Si-, and O-dominated particles with low contrast in the water. Flaky kaolinite (Al-Si-O) and diaspore (Al-O-H) can be observed as isolated particles (ID17-ID18). These clay minerals often form aggregates with other particles, such as ID12, in which halloysite combines with Pb-Mn-Fe particles to form an aggregation. As shown in previous studies, these clay minerals are prevalent in natural environments [33,41,42]. In summary, the particles in groundwater near the Mingzhu Industrial Park are thought to originate from factories, pipes, and natural sources. The types of elements and combinations of elements in the nanoparticles can reflect the macroscopic matter in the region.

### 4.2. Existing form and changes of nanoparticles

In our observations of nanoparticles from various sources, we identified several common characteristics. First, many nanoparticles, such as ID5, ID7, ID11, ID12, ID13, and ID15, exhibited aggregation. Nanoparticles often have a large specific surface area, which contributes to their strong adsorption ability [54,55] and facilitates their aggregation. It has been found that Ti can be transported in the form of particles in rivers [24] and some nanoparticles such as ZnO can migration in the aquifer porous media [56] and being stable in groundwater [57]. The particle ID13 in our study is an aggregate of Ti-W particles and halloysite, which directly reflects the adsorption and aggregation of Ti particles during transport. A similar aggregation phenomenon has been repeatedly observed in many particles in this study. Therefore, mutual adsorption and aggregation are common phenomena in particle transport. Additionally, the particles observed in the study tended to have a high O content. On one hand, the combination of nanoparticles and water molecules leads to a high O content in the test results; on the other hand, water has a high O content, and the nanoparticles are subject to significant oxidation [33,57], resulting in elevated oxygen levels. Oxidation is one of the chemical processes that nanoparticles can undergo in groundwater.

**Fig 6:**
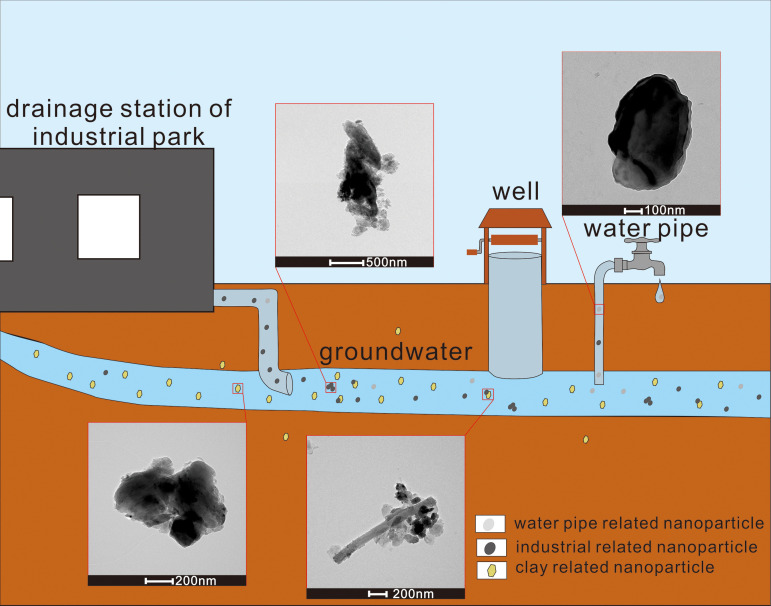
Schematic model illustrating the transport patterns of nanoparticles in groundwater near the industrial park.

In summary, based on the sources, transport patterns, and characteristics of the observed nanoparticles, a figure illustrating the transport pattern of nanoparticles in groundwater near the Mingzhu Industrial Park was created (Fig 6): During human activities at factories, large numbers of factory related nanoparticles are produced and transported through water bodies. Additionally, water pipes also generate nanoparticles due to the physical and chemical erosion caused by water [58–60]. The particles from the water pipes, those from the industrial park, and secondary mineral particles from the environment transport together, oxidizing and adsorbing each other in the process, thereby increasing their stability. The properties of nanoparticles can be influenced by many factories such as surrounding rocks, aquifer and environmental conditions, PH of aquifer, the presence or absence of organic matter and humic acid [56,57,61]. Ultimately, these nanoparticles remain stable in the current groundwater environment.

### 4.3. Possible environmental impacts of nanoparticles

Nanoparticles are one way these elements diffuse. Westerhoff et al. found that there are 895 ng/l Ti particles in the river, which account for 15.7% of the Ti content in the river water [24]. This indicates that nanoparticles represent a significant proportion of the pollutants transported. As a new type of pollutant, nanoparticles possess many characteristics. For example, they can be stable in groundwater and may cause harm to human health due to human activities. Due to their small size, nanoparticles can directly enter living organisms, including plants, animals, and even humans [26,53,62,63]. In this research, most nanoparticles are oxide nanoparticle which can produce excess generation of reactive oxygen species causing an imbalance in biological systems [64]. Inorganic nanoparticle, which was also found in this area, can cause cellular damage, dysfunction, oxidative stress, inflammation and damage multiple organs and systems, including the central nervous system [65.^66^,^67^]. Besides, the repeatedly detected ZnO nanoparticle in this area was proved previously to significantly affect the quality of the heart issue by damage the cardiomyocytes [68]. Once these nanoparticles enter the body, they may be difficult to eliminate and can accumulate within it [69,70]. This increases the risk of exposure and damage to surrounding tissues. Therefore, our discovery of nanoparticles in groundwater near industrial parks bridges laboratory toxicology and real-world environmental hazard assessment, enabling research on the environmental impact of nano pollutants. In addition, nanoparticles can transport long distances in rivers [71–72]. Zhou et al. Reported that nanoparticles can travel more than 7 km in surface water [73]. This property enhances the diffusion of heavy metal elements and increases the likelihood of heavy metal impacts around industrial parks. In conclusion, the presence of nanoparticles facilitates the transport of heavy metal particles over greater distances, allowing them to directly enter the human body and increasing their toxicity. Such particles are found not only near industrial parks but also in artificial environments, including household water pipes [44]. Therefore, the pollution caused by this large number of nanoparticles is very common and needs to be taken seriously [65]. Nanoparticulate pollution in areas such as the Mingzhu Industrial Park has not been taken seriously or dealt with. In recent years, methods such as coagulation-sedimentation, adsorption and ultrafiltration have been used to reduce nanoparticle content in water bodies in laboratory [74–77]. The properties, morphology and toxicity of nanoparticles were analyzed to demonstrate the ubiquity of nanoparticulate pollution, provide data basis for the establishment of pollutant treatment standards covering nanoparticles, provides relevant information for the quantitative application of laboratory nanoparticle removal method in natural environment.

## 5. Conclusion

The analysis of nanometer and near-nanometer particles in the groundwater near the Mingzhu Industrial Park was conducted, and the composition and characteristics of these particles were more directly understood using transmission electron microscopy. The groundwater contains irregular nanoparticles and aggregates with a size range of 38-1,212 nm, which are categorized into three groups based on their elemental composition: Zn-Fe nanoparticles, heavy metal (Pb, Mn, Cr) nanoparticles, and Al-Si-O clay particles. The study area uses galvanized steel pipes for water supply, and Fe-Zn nanoparticles may be generated by the physical impact and chemical dissolution corrosion of the pipes caused by water bodies. The heavy metal particles are mainly oxides or sulfates, which are deemed to originate from the Mingzhu Industrial Park. The types and combinations of heavy metal elements in these particles correspond well with common materials found in the park. Due to the high specific surface area and low stability of nanoparticles, they may adsorb and form aggregates during transportation. Additionally, there is a high content of O in the nanoparticles, indicating that oxidation also occurs during particle transport. Heavy metal nanoparticles are widespread in the groundwater near the Mingzhu Industrial Park. Considering that nanoparticles can directly enter the human body and cause damage to human tissues, as well as transport over long distances, the impact of industrial parks on nearby humans may exceed expectations. In order to establish relevant monitoring and solutions covering nanoparticulate pollutants, more attention needs to be paid to the properties of nanoparticles particularly heavy metal nanoparticles.

## Supporting information

S1 Fig TEM image and HRTEM image of ID1. (RAR)

S2 Fig TEM image and SAED image of ID2. (RAR)

S3 Fig TEM image and SAED image of ID3. (RAR)

S4 Fig TEM image and HRTEM image of ID4. (RAR)

S5 Fig TEM image and SAED image of ID5. (RAR)

S6 Fig TEM image and SAED image of ID6. (RAR)

S7 Fig TEM image and SAED image of ID7. (RAR)

S8 Fig TEM image and HRTEM image of ID8. (RAR)

S9 Fig TEM image and HRTEM image of ID9. (RAR)

S10 Fig TEM image and SAED image of ID10. (RAR)

S11 Fig TEM image and HRTEM image of ID11. (RAR)

S12 Fig TEM image and SAED image of ID12. (RAR)

S13 Fig TEM image and HRTEM image of ID13. (RAR)

S14 Fig TEM image and HRTEM image of ID14. (RAR)

S15 Fig TEM image and HRTEM image of ID15. (RAR)

S16 Fig TEM image and HRTEM image of ID16. (RAR)

S17 Fig TEM image and SAED image of ID17. (RAR)

S18 Fig TEM image and SAED image of ID18. (RAR)

S1 Table EDS result of ID1. (RAR)

S2 Table EDS result of ID2. (RAR)

S4 Table EDS result of ID4. (RAR)

S3 Table EDS result of ID3. (RAR)

S5 Table EDS result of ID5. (RAR)

S6 Table EDS result of ID6. (RAR)

S7 Table EDS result of ID7. (RAR)

S8 Table EDS result of ID8. (RAR)

S9 Table EDS result of ID9. (RAR)

S10 Table EDS result of ID10. (RAR)

S11 Table EDS result of ID11. (RAR)

S12 Table EDS result of ID12. (RAR)

S13 Table EDS result of ID13. (RAR)

S14 Table EDS result of ID14. (RAR)

S15 Table EDS result of ID15. (RAR)

S16 Table EDS result of ID16. (RAR)

S17 Table EDS result of ID17. (RAR)

S18 Table EDS result of ID18. (RAR)
